# SILAC based protein profiling data of MKK3 knockout mouse embryonic fibroblasts

**DOI:** 10.1016/j.dib.2016.02.034

**Published:** 2016-03-02

**Authors:** Anup Srivastava, Amanda S. Shinn, TuKiet T. Lam, Patty J. Lee, Praveen Mannam

**Affiliations:** a Pulmonary, Critical Care and Sleep Medicine, Department of Internal Medicine, Yale University School of Medicine, New Haven, CT 06520-8057, USA; b MS & Proteomics Resource at Yale University, WM Keck Foundation Biotechnology Resource Laboratory, Department of Molecular Biophysics and Biochemistry, New Haven, CT 06520-8057, USA

**Keywords:** MKK3, Mitochondria, Proteomics, SILAC

## Abstract

This data article reports changes in the phospho and total proteome of MKK3 knock out (MKK3^−^^/^^−^) mouse embryonic fibroblasts (MEFs). The dataset generated highlights the changes at protein level which can be helpful for understanding targets of the MAP kinase signaling pathway. Data was collected after TiO_2_-based phosphopeptide enrichment of whole cell lysate at baseline condition with bottom-up SILAC-based LC MS/MS quantitative mass spectrometry. We report all the proteins and peptides identified and quantified in MKK3^−/−^ and WT MEFs. The altered pathways in MKK3^−/−^ MEFs were analyzed by Database for Annotation, Visualization and Integrated Discovery (DAVID, v6.7) and Ingenuity Pathway Analysis (IPA) and are presented as a table and graph, respectively. The data reported here is related to the published work [1]. All the associated mass spectrometry data has been deposited in the Yale Protein Expression Database (YPED) with the web-link to the data: http://yped.med.yale.edu/repository/ViewSeriesMenu.do;jsessionid=6A5CB07543D8B529FAE8C3FCFE29471D?series_id=5044&series_name=MMK3+Deletion+in+MEFs.

**Specifications Table**

**Table:** 

Subject area	*Cell Biology and Kinase signaling*
More specific subject area	*MAP kinase signaling*
Type of data	*Table and graph*
How data was acquired	*LC MS/MS, bottom-up approach with CID in the ion trap. Peptides were separated on a Waters nanoACQUITY (column: 75 µm×250 mm eluted at 300 nl/min.; 80 min run) with MS analysis on an Orbitrap Elite mass spectrometer, Thermo Fisher.*
Data format	*Filtered by Proteome Discoverer v1.4, and database searched via in-house MASCOT Search Engine.*
Experimental factors	*Effect of MKK3 deletion on the proteome of mouse embryonic fibroblasts*
Experimental features	*Baseline difference in MKK3 knockout and wild-type mouse embryonic fibroblasts*
Data source location	*New Haven, CT, USA.*
Data accessibility	*Data available within this article and at*: http://yped.med.yale.edu/repository/ViewSeriesMenu.do;jsessionid=6A5CB07543D8B529FAE8C3FCFE29471D?series_id=5044&series_name=MMK3+Deletion+in+MEFs

**Value of the data**

• Proteomic characterization of the effect of MKK3 deletion, which provides data on MAP kinase signaling targets [1], [2], [3], [4], [5].
• The phospho and total protein change data has been presented as a reference for other investigators to check other signaling molecules in MAP kinase pathway.
• The major canonical pathways affected in MKK3 deleted MEFs are tabulated and can be utilized to interrogate the role of MKK3 in other signaling schemes.

## 1 Data

**Fig. 1:**
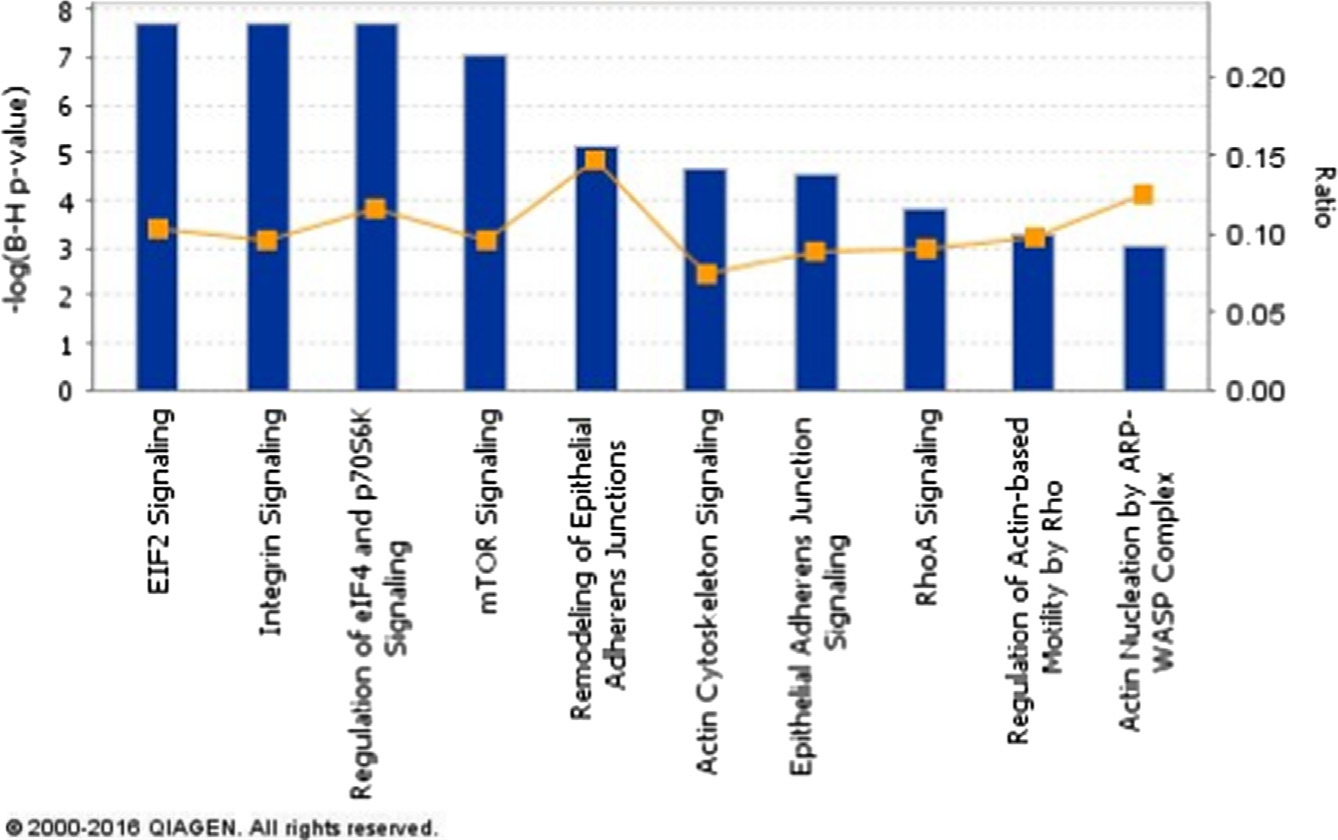
List of Canonical pathways affected by MKK3 deletion. The proteins increased or decreased by 30% were utilized to generate affected canonical pathways by IPA. Benjamin–Hochberg Multiple testing correction was applied for the analysis.

In this paper we provide the data generated from MKK3^−/−^ MEFs, reflecting the signaling pathways affected by MKK3. We included one table and one graph, which were generated by pooling all the proteins increased or decreased by 30% and analyzing them with DAVID [6], [7] and QIAGEN’s Ingenuity® Pathway Analysis (IPA®, QIAGEN Redwood City), respectively. The DAVID analysis of the data reflects changes in the processes up regulated by MKK3 with the threshold criteria of at least five genes affected (Table 1). IPA analysis depicts the top canonical pathways affected with the search criteria having Benjamin–Hochberg Multiple testing correction (Fig. 1).

## 2 Experimental design, materials and methods

**Fig. 2:**
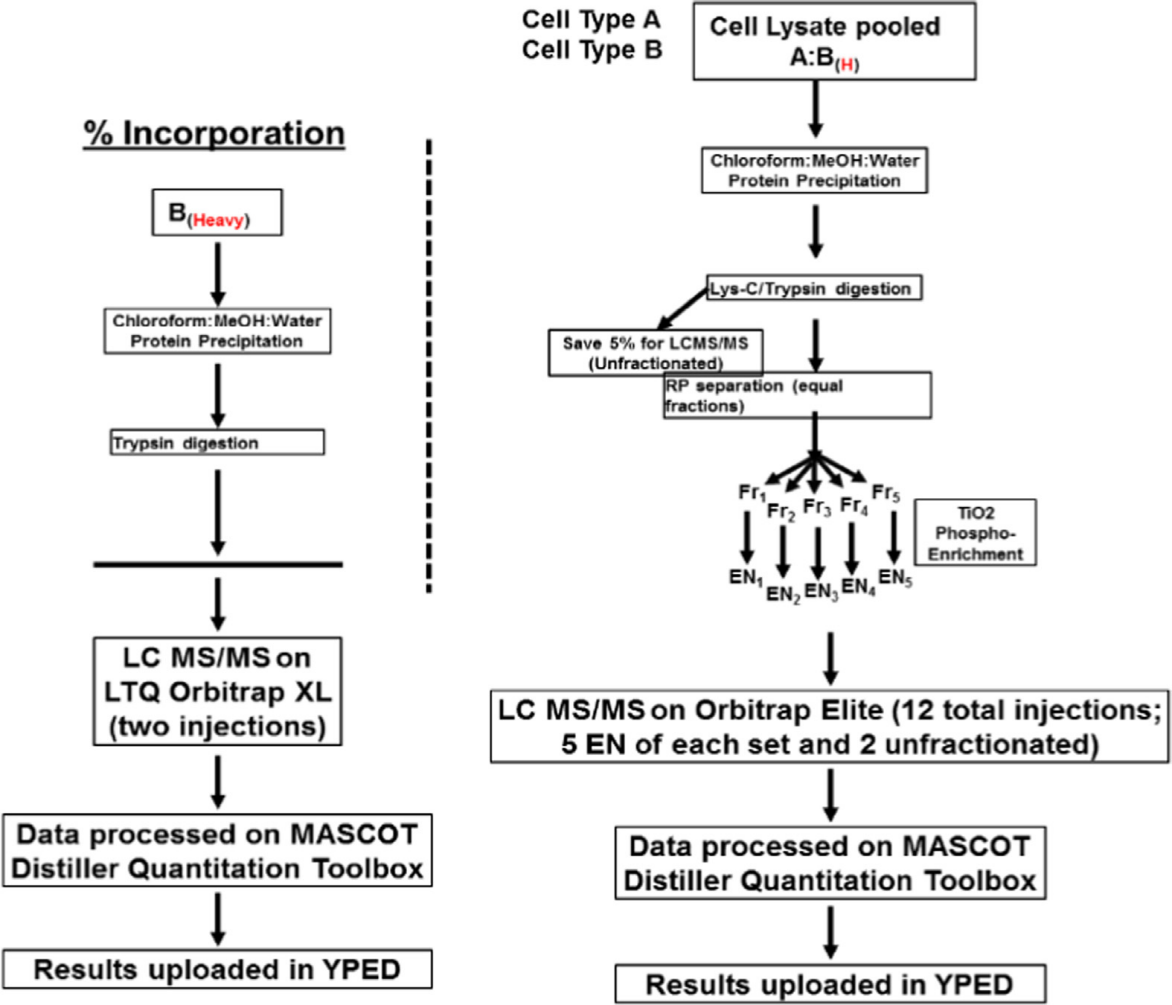
The workflow scheme of SILAC data generation.

This report summarizes the LC–MS/MS data acquired after trypsin digestion of cell lysate proteins which were labeled by SILAC for 5 passages. The scheme of the workflow is depicted in Fig. 2. Peptides were separated on a Waters nanoACQUITY (75 µm×250 mm eluted at 300 nl/min; 80 min run) with MS analysis on an Orbitrap Elite mass spectrometer.

### 2.1 Database searching and criteria

Mascot Distiller and the Mascot search algorithm were used for database searching (see Matrix Science for details, [8]) with following criteria: Database: SwissProt 2013_12; Taxonomy: *Mus musculus*, Enzyme: Trypsin/P; Max missed cleavages: 2; Variable modifications: Carbamidomethyl (C), Oxidation (M), Label: 13C (6) (K), Label: 13C (6)15N (4) (R); Peptide mass tolerance: ±10 ppm; Fragment mass tolerance: ±0.5 Da. Confidence level was set to 95% within the MASCOT search engine for protein hits based on randomness.

### 2.2 Identification of proteins

2 or more MS/MS spectra match were performed for the same protein entry in the database. The matched peptides were derived from the type of enzymatic digestion performed on the protein. Proteins listed as matching with only 1 significant peptide were not considered positive identifications. Some of the proteins that contain exactly the same peptides but different accession numbers are listed as indistinguishable meaning we cannot differentiate between these accession numbers. Other proteins may be present in the sample that were not identified by this analysis due to dynamic range issues in the mass spectrometer.

### 2.3 Functional annotation chart preparation

Proteins changed by at least 30% were identified. This list was loaded on DAVID with a mouse background. The protein list was analyzed by functional annotation tool and affected pathways were analyzed by KEGG Pathways. The search criteria included a minimum of 5 count and EASE score of 0.01. There were 11 pathways affected in MKK3^−/−^ MEFs compared to WT (Table 1).

### 2.4 Canonical pathway identification

The protein data was uploaded on IPA and pathway analysis performed. The reference set was IPA knowledge base, all data sources in IPA were included for analysis such as protein–protein interactions and microRNA–mRNA interactions. Only those relationships were considered where confidence was equal to experimentally observed. Stringent criteria was applied for filtering the results. B–H multiple testing correction *p*-value scoring method was used and values greater than 3 with a threshold value of 0.05 are displayed (Fig. 1).

## Figures and Tables

**Table 1:** Processes up regulated in MKK3^−^^/^^−^ MEFs**.** The data was analyzed by DAVID software and KEGG functional Annotation Chart is presented. The thresholds for count and EASE were 5 and 0.01, respectively.

**Term**	**Gene count**	**Fold enrichment**	***P*-value**	**Bonferroni**	**Benjamini**	**FDR**	**Fisher Exact**
Ribosome	25	6.5	1.30E−13	1.80E−11	1.80E−11	1.60E−10	1.50E−14
Citrate cycle (TCA cycle)	11	8.2	3.50E−07	4.80E−05	1.60E−05	4.10E−04	3.00E−08
Focal adhesion	27	3.2	2.40E−07	3.30E−05	1.70E−05	2.80E−04	6.80E−08
Regulation of actin cytoskeleton	24	2.6	4.60E−05	6.30E−03	1.60E−03	5.40E−02	1.60E−05
Tight junction	17	2.9	1.90E−04	2.60E−02	5.20E−03	2.20E−01	5.80E−05
Pyruvate metabolism	9	5.1	2.80E−04	3.80E−02	6.50E−03	3.30E−01	4.60E−05
Adherens junction	11	3.4	1.40E−03	1.70E−01	2.70E−02	1.60E+00	3.60E−04
Aminoacyl-tRNA biosynthesis	8	4.4	1.80E−03	2.20E−01	2.80E−02	2.10E+00	3.50E−04
Leukocyte transendothelial migration	14	2.7	1.60E−03	2.00E−01	2.80E−02	1.90E+00	5.40E−04
Glycolysis/gluconeogenesis	10	3.4	2.30E−03	2.70E−01	3.10E−02	2.60E+00	5.80E−04
Proteasome	8	4	3.50E−03	3.90E−01	4.30E−02	4.10E+00	7.70E−04
Arginine and proline metabolism	8	3.5	7.00E−03	6.20E−01	7.20E−02	7.90E+00	1.70E−03
Viral myocarditis	11	2.7	6.60E−03	6.00E−01	7.40E−02	7.50E+00	2.20E−03
